# Plasma versus autologous whole blood in the treatment of lateral epicondylitis: A systematic review and meta-analysis of randomized clinical trials

**DOI:** 10.1016/j.jcot.2026.103568

**Published:** 2026-07-16

**Authors:** Silvio Stafi Filho, Isabeli Comby, Francisney Pinto Nascimento

**Affiliations:** a Medical School, Universidad Central del Paraguay, Ciudad del Este, Paraguay; b Medical School, Universidade Federal da Integração Latino-Americana, Foz do Iguaçu, Brazil

**Keywords:** Tendinopathy, Elbow injuries, Biological therapy, Pain management, Rehabilitation

## Abstract

**Background:**

To compare platelet-rich plasma and autologous whole blood in adults with lateral epicondylitis, evaluating functional improvement and pain reduction across follow-up periods.

**Methods:**

Randomized clinical trials enrolling adults with clinically confirmed lateral epicondylitis and a minimum symptom duration of three months were systematically identified by searching PubMed, ScienceDirect, and the Cochrane Central Register of Controlled Trials. Functional improvement was prespecified as the primary outcome, whereas pain was the secondary outcome. Pooled effect estimates were obtained using random-effects meta-analyses conducted with Review Manager (RevMan) version 5.4.1, and between-study heterogeneity was quantified using the I^2^ statistic. Sensitivity analyses were performed to assess robustness in scenarios of substantial heterogeneity or when individual trials exerted excessive influence. The study protocol was prospectively registered in PROSPERO (CRD420250655318).

**Results:**

Four randomized clinical trials involving 322 participants were included, with 167 receiving platelet-rich plasma. Follow-up ranged from 1 to 12 months for function and 1 to 6 months for pain. No significant differences were observed between platelet-rich plasma and autologous whole blood for function, although a non-significant early trend favoring platelet-rich plasma was not sustained over time, with heterogeneity between 11% and 67%. Subgroup analysis showed no heterogeneity across follow-up durations (χ^2^ = 1.96; df = 3; p = 0.58; I^2^ = 0%). Pain reduction appeared greater with platelet-rich plasma when all studies were pooled; however, this difference disappeared after exclusion of a single high-risk-of-bias study, with heterogeneity decreasing to null levels except for low heterogeneity (25%) at six months. This indicates that pain outcomes were sensitive to an individual trial.

**Conclusion:**

Platelet-rich plasma did not demonstrate statistically significant superiority over autologous whole blood for functional outcomes, although a non-significant tendency favoring platelet-rich plasma was observed in early follow-up. Given the limited number of included studies, these findings should be interpreted with caution and considered exploratory.

## 1 Introduction

Lateral epicondylitis is a chronic degenerative tendinopathy involving the origin of the common extensor tendon at the lateral epicondyle of the humerus and is clinically manifested by persistent lateral elbow pain accompanied by reduced grip strength.^1^, ^2^, ^3^ It is widely recognized as an overuse-related disorder in both sports medicine and occupational contexts, particularly affecting individuals exposed to repetitive wrist-extension activities.^1^^,^^3^ Although its prevalence in the general population is estimated to range between 1% and 3%, the condition imposes a substantial socioeconomic burden, contributing to work absenteeism, decreased productivity, and impairment in quality of life.^1^^,^^2^ Histopathological evidence indicates that lateral epicondylitis is characterized by angiofibroblastic tendinosis rather than a primarily inflammatory process. Findings include collagen fiber disorganization, increased fibroblastic proliferation, and neovascularization, all of which are implicated in sustained pain and functional limitation.^3^

Conservative treatment is considered the standard initial approach for lateral epicondylitis, primarily based on physical therapy, activity modification, and pharmacological pain control.^1^ In cases of persistent symptoms, injectable biological therapies have emerged as therapeutic alternatives to corticosteroid injections and surgical intervention in refractory cases of lateral epicondylitis.^4^ Among these approaches, platelet-rich plasma (PRP) and autologous whole blood (AWB) have been increasingly applied with the objective of promoting tendon repair through the local delivery of bioactive growth factors.^4^, ^5^, ^6^, ^7^ Both PRP and AWB may modulate inflammatory and analgesic pathways by attenuating the release of pro-algesic mediators involved in nociceptive signaling, including prostaglandins, nitric oxide, bradykinin, histamine, serotonin, and cytokines, thereby potentially facilitating tissue regeneration.^6^, ^7^, ^8^, ^9^, ^10^ However, despite these shared mechanisms, differences in the biological composition of PRP and autologous whole blood, particularly regarding erythrocyte content, may influence the local tissue environment and potentially impact clinical outcomes.^11^

According to regulatory guidelines issued by European, American, Australian, and Japanese authorities, current regulation is limited to ensuring product quality, safety, and traceability, without addressing minimum dosing requirements or the number of applications necessary to achieve therapeutic efficacy in tendinopathies.^12^, ^13^, ^14^, ^15^, ^16^ This lack of standardization may contribute to variability in clinical outcomes and should be considered when interpreting the available evidence.

Several randomized clinical trials have compared PRP and AWB, but results remain inconsistent. While some studies report superior functional recovery with PRP, others have found no significant differences between the interventions regarding pain reduction or long-term outcomes.^17^^,^^18^ Network meta-analyses and systematic reviews have also yielded heterogeneous conclusions, partly due to variability in PRP preparation, application protocols, and follow-up periods.^19^, ^20^, ^21^

Given these inconsistencies, the optimal choice between PRP and AWB for the treatment of lateral epicondylitis remains uncertain. Given their increasing clinical use and the lack of consensus on comparative efficacy, we conducted a systematic review and meta-analysis of randomized clinical trials directly comparing PRP and AWB in lateral epicondylitis. We evaluated both functional improvement and pain relief. Separate analyses were performed for different follow-up periods. This study was conducted and reported in accordance with the Cochrane Handbook for Systematic Reviews of Interventions^22^ and the PRISMA 2020 guidelines.^23^

## 2 Methods

### 2.1 Eligibility criteria

Studies were considered eligible if they met the following criteria: (1) randomized clinical trial (RCT); (2) direct comparison between platelet-rich plasma and autologous whole blood; (3) adult participants with a clinically confirmed diagnosis of lateral epicondylitis based on compatible medical history and physical examination; and (4) symptom duration of at least three months. Eligible studies were required to report at least one predefined clinical outcome related to function and/or pain, assessed using validated instruments with extractable data suitable for quantitative synthesis. Variations in PRP preparation and administration were accepted, including differences in platelet concentration, activation method, injected volume, number of applications, and use of local anesthetics. Studies were excluded if they lacked a control group, did not directly compare PRP with AWB, or included participants without a confirmed diagnosis of lateral epicondylitis. In cases of overlapping populations, the study with the largest sample size was included. No restrictions were applied regarding language, publication type, or publication status.

### 2.2 Search strategy

A comprehensive literature search was conducted in PubMed, ScienceDirect, and the Cochrane Central Register of Controlled Trials (CENTRAL), including records from database inception through July 2025. The search strategy incorporated the following terms: (“platelet rich plasma” OR PRP) AND (“autologous blood” OR “whole blood”) AND (“lateral epicondylitis” OR “tennis elbow” OR “elbow tendinopathy” OR “elbow pain”). To ensure a thorough identification of relevant studies, the reference lists of all included trials, as well as those of pertinent systematic reviews and meta-analyses, were manually reviewed to identify additional eligible articles. Study selection and data extraction were independently performed by two reviewers. The review protocol was prospectively registered in PROSPERO on February 24, 2025 (CRD420250655318).

### 2.3 Outcomes

The primary outcome of this review was functional improvement, while pain intensity was considered the secondary outcome. Functional outcomes were evaluated using different validated instruments across the included trials (Table 1) and were pooled as standardized mean differences (SMD, Hedges’ g) with corresponding 95% confidence intervals (CI). Pain outcomes, which were consistently assessed using a 0–10 visual analog scale (VAS), were synthesized as mean differences (MD) with 95% CI. To allow direct comparison among functional measures, all scales were standardized so that higher scores represented worse functional status. This was performed in accordance with methodological guidance outlined in the Cochrane Handbook for Systematic Reviews of Interventions.^22^ Accordingly, the Mayo Elbow Performance Score in Raeissadat et al.^17^ and the Liverpool Elbow Score in Thanasas et al.^24^ were inverted before analysis. Because the Liverpool Elbow Score incorporates both objective and subjective domains with weighted components that are not directly comparable to other predominantly patient-reported instruments, the study by Thanasas et al.^24^ was excluded from the primary analyses of both outcomes due to substantial heterogeneity in the outcome measure, which limited comparability with the other included studies. Full analyses including this study are provided in the supplementary material, preserving methodological consistency in the primary analysis while allowing assessment of its influence. In the trial conducted by Creaney et al.,^25^ the standard deviation for age was not reported. The corresponding author was contacted; however, no response was obtained. To avoid unsupported assumptions, no imputation was performed, and the reported values were retained.

**Table 1:** – Baseline characteristics of the included studies.

Study	N randomized (PRP/AWB)	N analyzed (PRP/AWB)	Age^†^ (years) (PRP/AWB)	Female % (n) (PRP/AWB)	Right arm (%)	Symptom duration (months)	Injection volume (mL)	Baseline platelet count (L or μL)	Platelet conc. (×baseline)	Functional scale	Follow-up (months)
Creaney et al., 2011	80/70	70/60	53/48^a^	43% (34)/44% (31)^b^	NA	>6	3	6	234 × 10^9^/L	PRTEE	6
Linnanmäki et al., 2020	40/40	31/38	46 ± 5/46 ± 10	55% (22)/50% (20)	73/78	>3	4–6	12	NA	QuickDASH	12
Raeissadat et al., 2014	33/31	31/30	43 ± 6/44 ± 7	74% (23)/80% (24)	61/73	>3	2	12	250,000 ± 53,000/μL	Modified Mayo	12
Thanasas et al., 2011	14/14	14/14	35.9 (34–55)/36.6 (29–52)	71% (10)/79% (11)^b^	NA	≥3	3	6	NA	LES	6

a No standard deviation (SD) reported; † Values are presented as mean ± SD or as mean (range), according to the original publication.

b Female percentage and/or absolute numbers calculated from group sizes reported in the study; PRP: platelet-rich plasma; AWB: autologous whole blood; NA: not available; PRTEE: Patient-Rated Tennis Elbow Evaluation; QuickDASH: Quick Disabilities of the Arm, Shoulder and Hand; Modified Mayo: Modified Mayo Clinic Performance Index for the Elbow; LES: Liverpool Elbow Score.

### 2.4 Data synthesis and analysis

Meta-analytical comparisons were conducted according to each reported follow-up interval, including 1, 3, 6, and 12 months for functional outcomes and 1, 2, and 6 months for pain outcomes, in order to minimize the risk of unit-of-analysis errors. Follow-up intervals were defined after study inclusion because few trials reported identical time points, precluding analyses at uniform intervals. To evaluate the robustness of the pooled estimates and to identify potential sources of heterogeneity, leave-one-out sensitivity analyses were performed by sequentially excluding individual studies from the meta-analyses.

### 2.5 Risk of bias assessment

Methodological quality of the included trials was evaluated using version 2 of the Cochrane Risk of Bias tool (RoB 2). The assessment was conducted independently by two reviewers, with any disagreements resolved through discussion until consensus was reached. Risk of bias judgments were presented by individual domains and summarized using graphical representations.

### 2.6 Statistical methods

This systematic review was conducted in accordance with the Cochrane Handbook for Systematic Reviews of Interventions and reported following the PRISMA 2020 guidelines.^22^^,^^23^ Quantitative syntheses were performed using random-effects meta-analyses based on the DerSimonian and Laird method.^22^ Statistical heterogeneity among studies was evaluated using Cochran's Q test and the I^2^ statistic, with p values < 0.10 and I^2^ values > 50% interpreted as indicating substantial heterogeneity. An assessment of publication bias was not undertaken because fewer than ten studies were available for each outcome.

### 2.7 Software

All statistical analyses were performed using Review Manager (RevMan), version 5.4.1 (The Cochrane Collaboration, 2020). WebPlotDigitizer, version 4.6 (Austin, Texas, USA), was used to extract numerical data from graphs reported by Creaney et al.25, with extractions independently performed by two reviewers.

## 3 Results

**Fig. 1:**
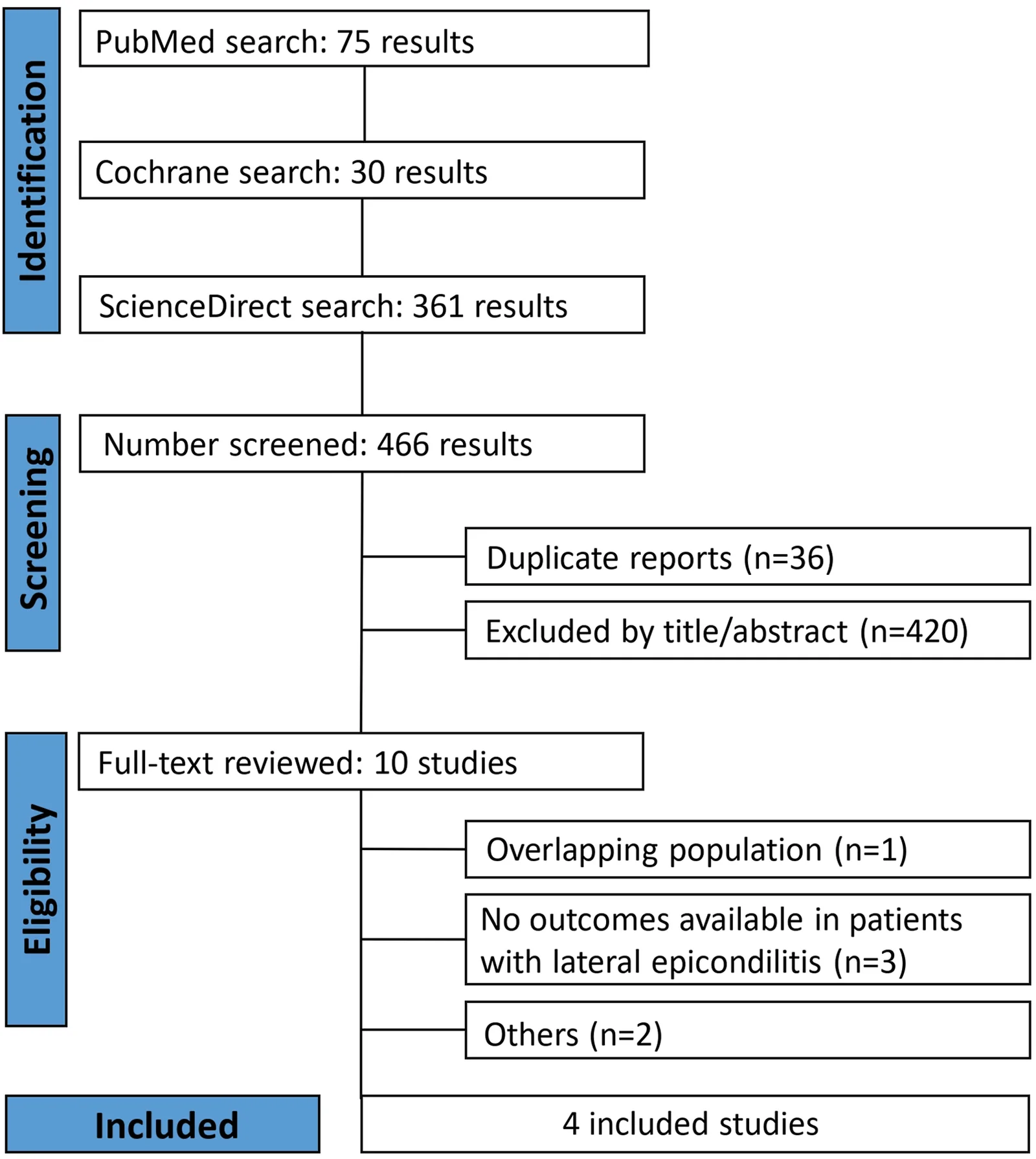
– PRISMA flow diagram of the study screening and selection process.

As detailed in Fig. 1, a total of 466 records were identified through database searches and manual screening of reference lists from relevant reviews and meta-analyses. After removing duplicates and unrelated studies, 10 articles were assessed in full text for eligibility. Four randomized clinical trials (RCTs) were included, comprising a total of 322 participants, of whom 167 were allocated to the PRP group. Baseline characteristics are summarized in Table 1. The trials were consistent regarding clinical confirmation of the diagnosis of lateral epicondylitis and a minimum follow-up of six months. All studies used validated instruments to assess function and pain, with variation in the scales chosen and in specific details of the injection protocols.

**Fig. 2:**
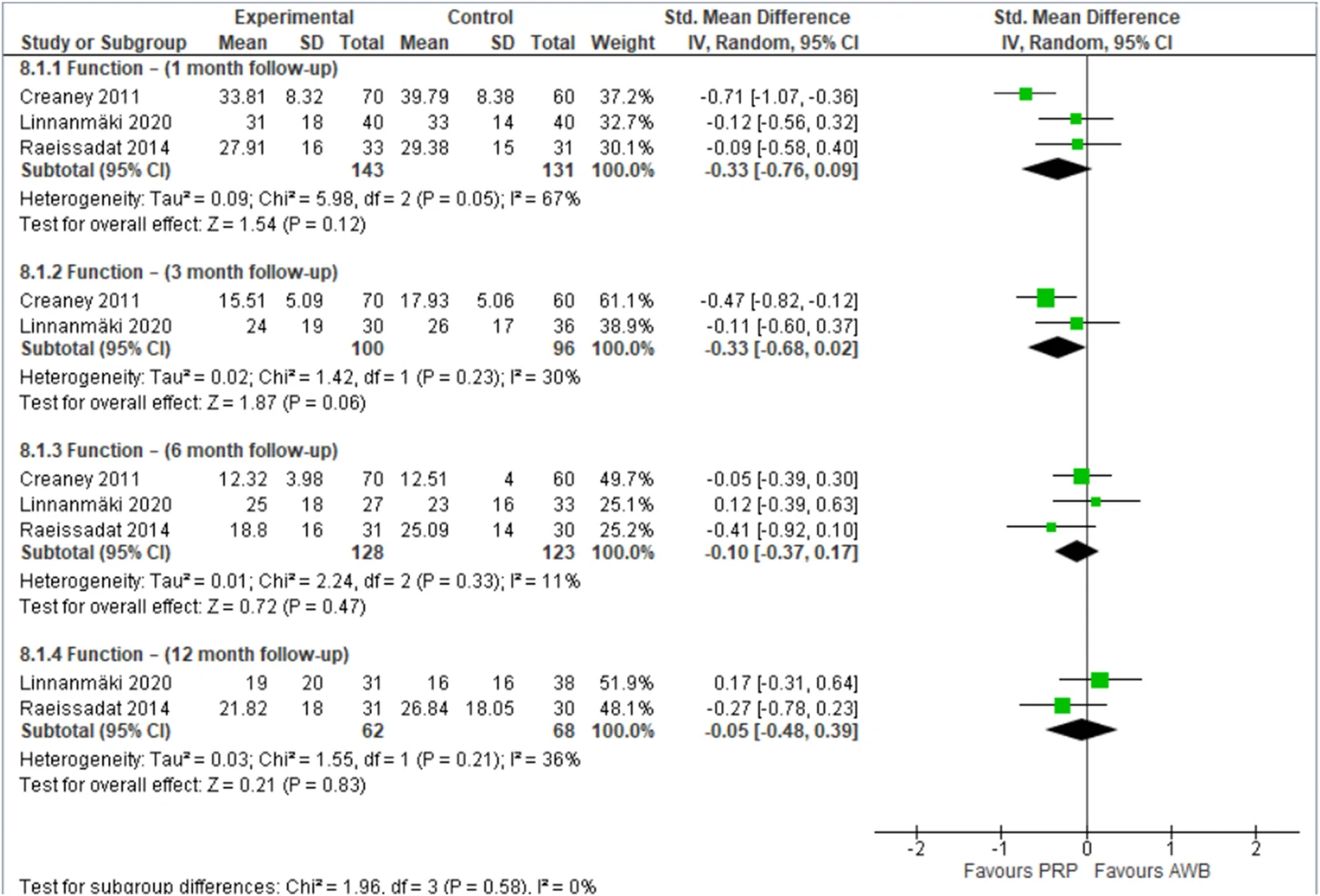
– Functional outcome analysis comparing PRP and AWB at different follow-up periods (1, 3, 6, and 12 months); effects showed a non-significant tendency favoring PRP in the early periods, with an effect approaching zero at 12 months. PRP: platelet-rich plasma; AWB: autologous whole blood.

Functional improvement showed numerically greater gains in the PRP group during the early follow-up periods, although the differences did not reach statistical significance, with an effect approaching zero at 12 months. Although not statistically significant, the direction of effect remained predominantly consistent during early follow-up, with I^2^ values ranging from 11% to 67% across analyses, indicating low to substantial heterogeneity (Fig. 2). The test for subgroup differences showed χ^2^ = 1.96, df = 3, p = 0.58, I^2^ = 0%, suggesting no heterogeneity between follow-up periods.

**Fig. 3:**
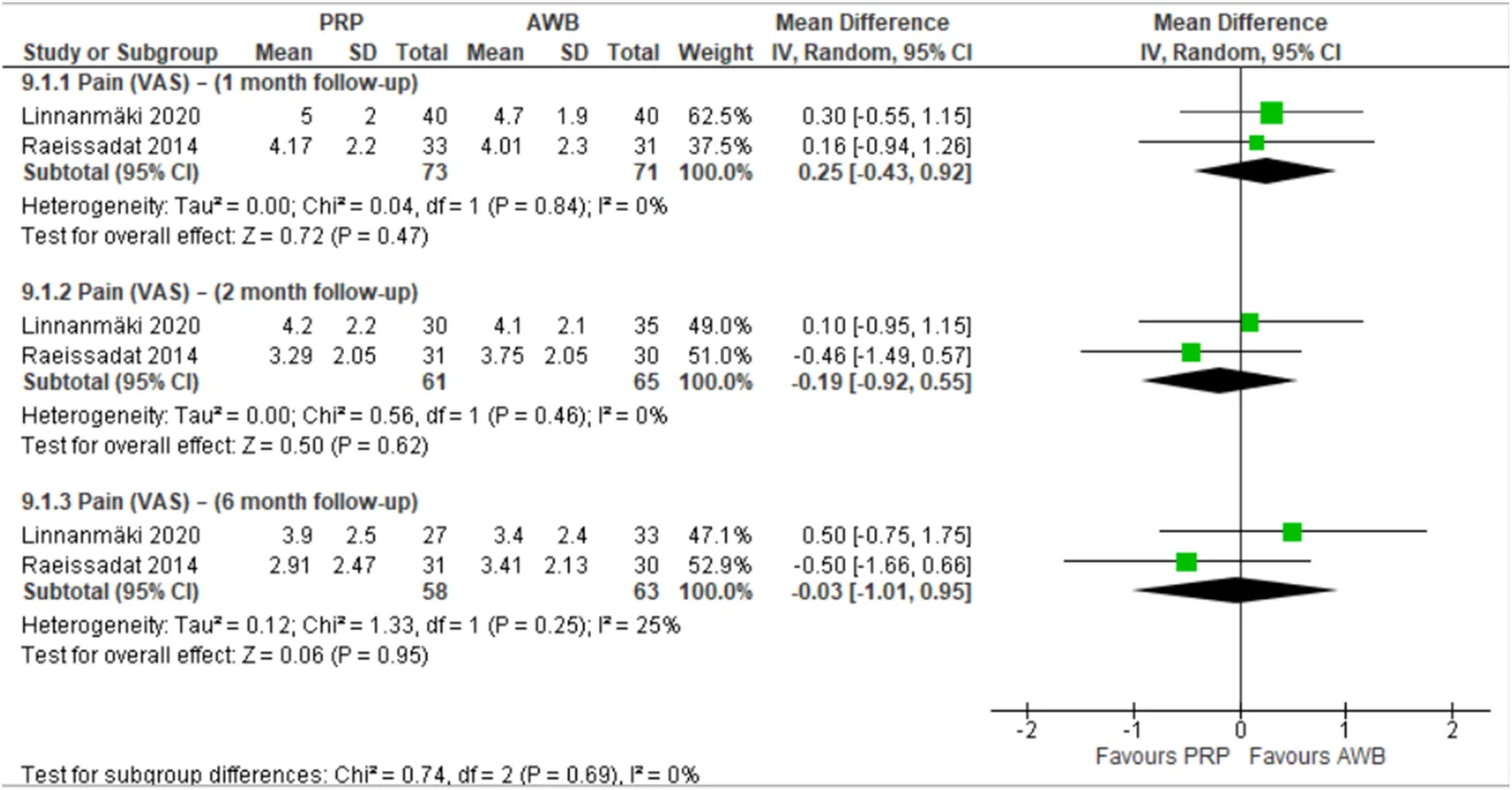
– Pain outcome analysis comparing PRP and AWB at different follow-up periods (1, 2, and 6 months); no significant difference was observed between groups, with null heterogeneity at 1 and 2 months and low heterogeneity at 6 months (25%). PRP: platelet-rich plasma; AWB: autologous whole blood.

Pain reduction was significantly greater with PRP when all studies were included, with heterogeneity ranging from 48% to 87% (Fig. S1). In the sensitivity analysis excluding Thanasas et al.24, no statistically significant difference was observed between PRP and AWB; heterogeneity was absent at 1 and 2 months and low at 6 months (I^2^ = 25%) (Fig. 3), with the effect estimates shifting toward the null. These findings indicate that the pain results were strongly influenced by Thanasas et al.,^24^ limiting the robustness of the pooled estimates and increasing uncertainty in their interpretation. Separate analyses by follow-up period (1, 3, 6, and 12 months for function; 1, 2, and 6 months for pain), including the study by Thanasas et al.,^24^ showed that pain outcomes remained non-significant but with substantial variation in heterogeneity across follow-up periods, while functional outcomes showed an early effect that diminished over time. In contrast, exclusion of this study resulted in more consistent estimates with lower heterogeneity across all time points. These findings indicate that inclusion of this trial introduces instability in pooled estimates, supporting its exclusion from the primary analysis while maintaining transparency through sensitivity analyses. Detailed analyses, including study-specific effects, are provided in the supplementary material (see Fig. S1).

**Fig. 4:**
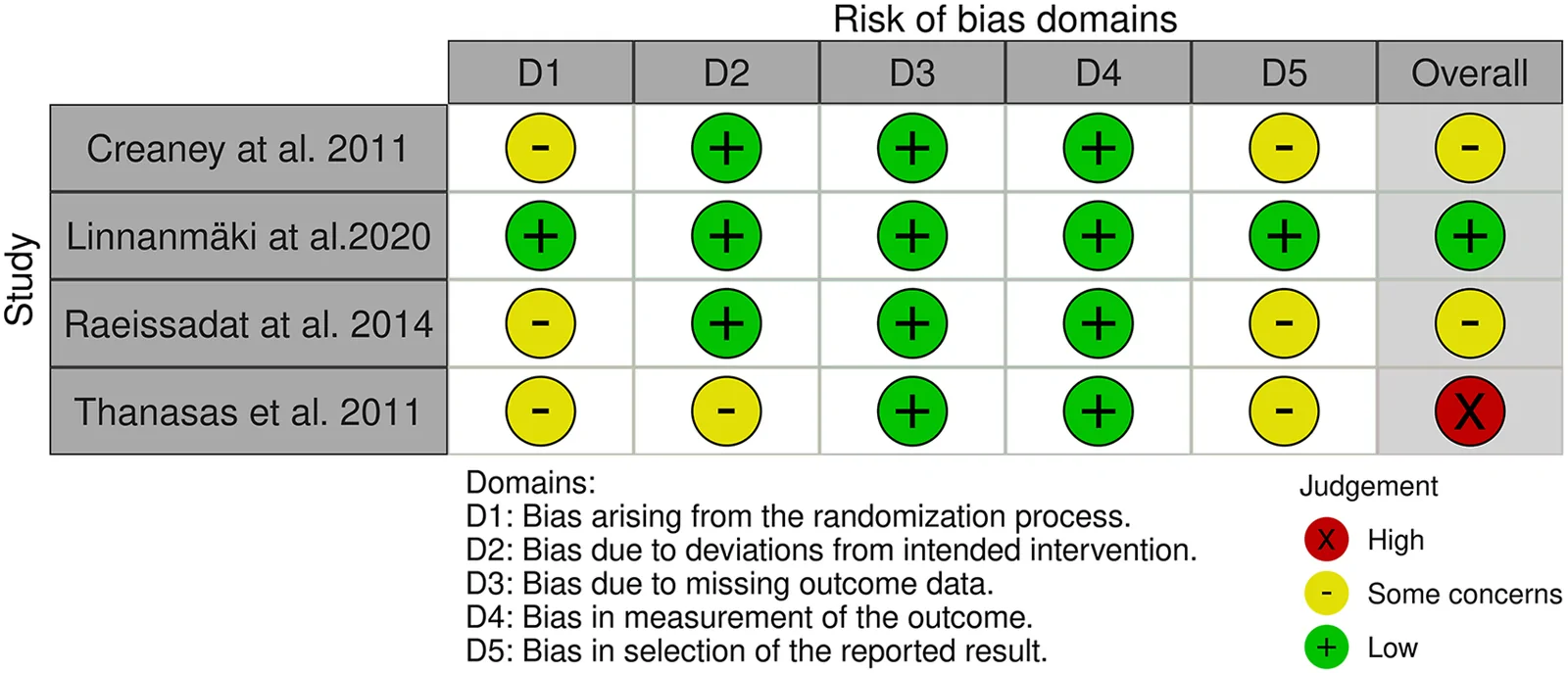
– Risk of bias assessment (RoB 2) of the included studies.

Risk of bias assessment is presented in Fig. 4. Three trials showed concerns related to the randomization process and/or lack of blinding, whereas Linnanmäki et al. (2020)^26^ was classified as low risk in all domains. Thanasas et al. (2011)^24^ was judged to have an overall high risk of bias due to limitations in the randomization process and deviations from intended interventions. Risk of bias assessment using the RoB 2 tool was conducted for both outcomes: the primary outcome (function) and the secondary outcome (pain). The latter is presented in the supplementary material, ensuring transparency and consistency with the methodology described.

## 4 Discussion

In this systematic review and meta-analysis of four randomized clinical trials involving 322 patients with lateral epicondylitis, we compared the efficacy of PRP and AWB in improving function and reducing pain. The main findings were: (1) functional outcomes were numerically superior in the PRP group during the early follow-up periods, without a statistically significant difference, with an effect approaching zero at 12 months; (2) pain reduction was significantly greater with PRP when all studies were included, but this difference was no longer significant after excluding the study by Thanasas et al.^24^; and (3) sensitivity analyses did not change the overall interpretation for function but substantially altered the results for pain, indicating that this outcome is strongly influenced by a single study, which limits the robustness of the pooled estimates and introduces uncertainty in the interpretation of the results, rendering the pain findings exploratory. Given the limited number of included trials, these findings should be interpreted with caution, as the statistical power is restricted and the robustness of pooled estimates may be affected.

The use of PRP for lateral epicondylitis has been widely investigated, with heterogeneous findings. Previous meta-analyses have evaluated similar interventions; however, they often included heterogeneous treatment comparisons, non-randomized designs, or broader intervention networks. In contrast, the present study included only randomized controlled trials directly comparing PRP and AWB in lateral epicondylitis, allowing for a more focused and internally consistent assessment of their comparative effects. In a network meta-analysis, Arirachakaran et al.^4^ found PRP and AWB to be superior to corticosteroids, whereas direct comparisons did not show consistent differences in function or pain. In addition, the limited number of included trials reflects the scarcity of recent randomized studies directly comparing PRP and AWB, highlighting the need for further high-quality trials in this field. Similarly, a systematic review and meta-analysis by Fitzpatrick et al.^27^ reported sustained functional benefits of PRP in chronic tendinopathies, including lateral epicondylitis, although without direct comparisons between PRP and AWB.

In the specific context of the direct comparison between PRP and AWB, our findings reinforce that, in our analysis, the effect estimates for PRP showed a similar pattern across follow-up periods, although without statistical significance for functional outcomes. This may be related to its regenerative potential and modulation of the local inflammatory response, whereas analgesia may depend on additional factors.^6^^,^^7^

The high autologous platelet concentration in PRP enhances the release of these bioactive molecules. In addition, PRP promotes tenocyte and fibroblast proliferation, increasing type I and III collagen synthesis, and exerting an anti-inflammatory effect mediated by HGF, which inhibits the NF-κB pathway, contributing to tissue regeneration.^27^

Autologous whole blood has been used based on the premise that injection into the affected tendon may stimulate a local healing response.^10^^,^^19^ It contains lower physiological platelet concentrations, but includes similar biological mediators, resulting in a more gradual inflammatory response.^18^^,^^27^ After application, a local microtrauma may trigger a controlled inflammatory response mediated by platelets and leukocytes, with recruitment of immune cells, increased vascular permeability, and reactivation of the healing cascade in degenerative tendons.^27^ In addition, AWB contains other cellular components, particularly erythrocytes, which may influence the local tissue environment.^11^ Although AWB provides biological mediators similar to PRP, erythrocytes may not be biologically inert. Emerging evidence suggests that erythrocyte lysis during or after application may release free hemoglobin, iron, and heme, associated with oxidative stress, activation of inflammatory pathways, and direct cytotoxic effects on musculoskeletal tissues, negatively impacting tissue regeneration.^11^ In this context, although AWB may induce a controlled inflammatory response mediated by platelets and leukocytes, the presence of erythrocytes may represent a limiting or potentially detrimental factor in tendon healing.^11^

This distinction is relevant to the interpretation of our findings, as PRP is designed to concentrate platelets and growth factors while reducing erythrocytes.^6^^,^^7^ Therefore, differences between PRP and AWB may reflect not only variations in platelet concentration, but also fundamental differences in the biological composition of the injected material. Accordingly, our findings should be interpreted not only as a comparison of two autologous therapies, but also of distinct biological environments, which may partially explain the variability observed across studies.

A directional pattern favoring PRP was observed across different follow-up periods in our analysis and may suggest a possible early effect, although this was not statistically significant and was not sustained over time, particularly in the early phases (1–3 months). This is consistent with Raeissadat et al.,^17^ in which PRP produced greater functional gains at three months, with partial maintenance at six months. In contrast, Linnanmäki et al.^26^ found no significant differences between PRP and AWB at 12 months, highlighting the importance of follow-up duration as a critical variable in interpreting outcomes.

The absence of significant differences in pain reduction between PRP and AWB, also reported in previous studies, may reflect overlapping analgesic mechanisms between the two therapies, as both involve local infiltration of autologous blood and a controlled inflammatory stimulus.^17^^,^^26^ Although PRP contains higher concentrations of growth factors, the nociceptive response may be influenced by interindividual variability, injection technique, and number of applications, which may attenuate differences in pain scores when compared with AWB.^6^^,^^7^

In addition to the biological differences between PRP and AWB, an important methodological factor is the heterogeneity of PRP formulations across studies. Prior evidence demonstrates substantial variability in PRP preparation, including differences in centrifugation protocols, commercial systems, activation strategies, and injected volumes, with multiple preparation approaches described in the literature.^18^ Importantly, many studies do not adequately characterize PRP composition, and only a minority report key parameters such as platelet concentration and leukocyte content.^18^^,^^28^ This variability may have contributed to the heterogeneity observed in the pooled analyses and to the lack of consistent treatment effects across studies.

From a biological standpoint, PRP is not a uniform intervention, as its effects depend on the concentration of platelets, growth factors, and cellular components delivered to the target tissue^6^˒.^7^ Clinical evidence also indicates that outcomes may vary according to PRP preparation characteristics. Fitzpatrick et al.^27^ reported differences in outcomes across PRP types, with more favorable results in studies using leukocyte-rich, highly cellular preparations, while emphasizing the importance of preparation method and injection technique.^27^^,^^29^

In our analysis, Table 2 demonstrates variability among the included trials in platelet concentration, leukocyte classification, activation status, anticoagulant use, and processed blood volume, while platelet dose was not reported in any study. Therefore, the absence of consistent superiority of PRP over AWB in our meta-analysis should be interpreted with caution, as it may reflect variability in PRP formulations rather than definitive equivalence between interventions. In this context, the absence of platelet dose reporting and variability in PRP preparation suggests that suboptimal dosing may have contributed to the lack of consistent superiority across studies. This is particularly relevant in our analysis, as variability in platelet concentration and the absence of dose reporting across all included trials limit the ability to ensure that a biologically adequate PRP dose was delivered.

**Table 2:** – PRP related parameters in the included studies.

Study	PRP preparation method	Leukocyte (count/content)	Platelet concentration (vs AWB)^a^	RBC content	Activator	Platelet dose	Blood volume processed (mL)	Anticoagulant
Creaney et al., 2011	Single spin	LR-PRP	2.8x	NA	NA	NA	8.5	Citrate
Linnanmäki et al., 2020	Single spin	LP-PRP	1.99x	NA	NA	NA	≈15	No anticoagulant
Raeissadat et al., 2014	Double spin	LR-PRP	4.8x	NA	NA	NA	20	ACD-A
Thanasas et al., 2011	Single spin	LR-PRP	5.5x	NA	No activator	NA	27–55	Not specified

a Platelet concentration (vs AWB) was extracted as reported in the original studies; PRP: platelet-rich plasma; AWB: autologous whole blood; NA: not available; LR-PRP: leukocyte-rich platelet-rich plasma; LP-PRP: leukocyte-poor platelet-rich plasma; RBC: red blood cells; ACD-A: acid citrate dextrose – solution A.

This study has several methodological strengths. The review protocol was prospectively registered, and the study was conducted in accordance with the Cochrane Handbook and PRISMA 2020 guidelines.^22^^,^^23^ A comprehensive search strategy was applied across multiple databases, and only randomized controlled trials directly comparing PRP and AWB were included, ensuring a focused and methodologically consistent analysis.

Our study has some limitations. The limited number of trials and participants constrains the statistical power for more robust subgroup analyses and limits the generalizability of the findings. Accordingly, the results should be interpreted with caution and considered exploratory rather than definitive. Further, to date, no official guideline defines a minimum effective dose. Regulatory agencies and European guidelines address quality and safety (regulation, preparation, traceability), but not minimum clinical dosing required for efficacy in tendinopathies in any document.^12^^,^^16^ PRP preparation and administration techniques varied in injected volume, activation methods, and number of applications, in addition to the inconsistent use of co-interventions (e.g., local anesthetic), which may have contributed to the current literature heterogeneity. Furthermore, three of the four included studies did not implement blinding, increasing the risk of performance bias, particularly for self-reported outcomes such as pain and function.^17^^,^^24^^,^^25^ Follow-up durations varied, and not all studies provided data for every interval. The disproportionate influence of the study by Thanasas et al.^24^ on the pain results represents a limitation, as it showed an effect direction opposite to that observed in the other trials, highlighting the sensitivity of the pooled estimates to individual studies. Differences in the functional scales used, even after standardization, may introduce residual variability. Additionally, the absence of complete baseline data for some variables (e.g., platelet count in part of the studies) prevents a more detailed analysis of prognostic factors.

## 5 Conclusion

This meta-analysis of 322 patients suggests that functional improvement from 1 to 12 months does not differ significantly between PRP and AWB, with no consistent or statistically significant superiority of PRP observed across studies, although early effect estimates suggested a possible direction favoring PRP that was not sustained over time, with an effect approaching zero at 12 months. The pooled pain estimates were sensitive to the inclusion of a single high-risk-of-bias study and should therefore be interpreted as exploratory. Given the limited number of included studies, the overall findings should be interpreted with caution. Further studies are needed to clarify the role of PRP and AWB in this condition.

## Guardian/patient's consent

Not applicable. This study is a systematic review and meta-analysis based exclusively on data from previously published studies. No individual patient data were collected, and no direct patient or guardian involvement occurred.

## Ethical statement

Ethical approval was not required for this study, as it is a systematic review and meta-analysis conducted using data from previously published studies. The review protocol was prospectively registered in PROSPERO (CRD420250655318), and the study was conducted in accordance with the Cochrane Handbook for Systematic Reviews of Interventions and the PRISMA 2020 guidelines.

## Author contributions

Silvio Stafi Filho: Conceptualization, Methodology, Formal analysis, Statistical analysis, Data curation, Investigation, Writing (original draft), Writing (review and editing), Visualization.

Isabeli Comby: Conceptualization, Methodology, Formal analysis, Data curation, Investigation, Writing (original draft), Writing (review and editing), Visualization.

Francisney Pinto Nascimento: Supervision, Methodology, Writing (original draft), Writing (review and editing), Project administration.

## Ethics

Not applicable (systematic review and meta-analysis of published studies only).

## Declaration of generative AI and AI-assisted technologies in the writing process

During manuscript preparation, the authors used ChatGPT (OpenAI) to assist with formatting, grammar, and structural organization. The authors reviewed and edited the content and take full responsibility for the final version.

## Declaration of generative AI and AI-assisted technologies in the writing process

The authors used ChatGPT solely for organizational assistance. All scientific content was produced and verified by the authors.

## Funding

This research did not receive any specific grant from funding agencies in the public, commercial, or not-for-profit sectors.

Declarations of interest: None.

## Declaration of competing interest

None.
