# Supplementary material for: Plasma versus autologous whole blood in the treatment of lateral epicondylitis: A systematic review and meta-analysis of randomized clinical trials
Source: J Clin Orthop Trauma. 2026 Jul 16;82:103568. doi: 10.1016/j.jcot.2026.103568 (PMC13396905; doi:10.1016/j.jcot.2026.103568)
Supplement: Supplementary file 1 [file mmc1.pdf]

Study or Subgroup	Experimental Mean	Experimental SD	Experimental Total	Control Mean	Control SD	Control Total	Weight	Std. Mean Difference IV, Random, 95% CI
**8.1.1 Function – (1 month follow-up)**								
Creaney 2011	33.81	8.32	70	39.79	8.38	60	28.8%	-0.71 [-1.07, -0.36]
Linnanmäki 2020	31	18	40	33	14	40	27.2%	-0.12 [-0.56, 0.32]
Raeissadat 2014	27.91	16	33	29.38	15	31	26.2%	-0.09 [-0.58, 0.40]
Thanasas 2011	0.99	0.19	14	1.32	0.14	14	17.8%	-1.92 [-2.84, -1.00]
**Subtotal (95% CI)**			**157**			**145**	**100.0%**	**-0.60 [-1.18, -0.03]**
Heterogeneity: Tau² = 0.27; Chi² = 16.01, df = 3 (P = 0.001); I² = 81%								
Test for overall effect: Z = 2.05 (P = 0.04)								
**8.1.2 Function – (3 month follow-up)**								
Creaney 2011	15.51	5.09	70	17.93	5.06	60	37.7%	-0.47 [-0.82, -0.12]
Linnanmäki 2020	24	19	30	26	17	36	35.7%	-0.11 [-0.60, 0.37]
Thanasas 2011	0.84	0.2	14	1.28	0.17	14	26.5%	-2.30 [-3.29, -1.32]
**Subtotal (95% CI)**			**114**			**110**	**100.0%**	**-0.83 [-1.70, 0.04]**
Heterogeneity: Tau² = 0.49; Chi² = 15.34, df = 2 (P = 0.0005); I² = 87%								
Test for overall effect: Z = 1.87 (P = 0.06)								
**8.1.3 Function – (6 month follow-up)**								
Creaney 2011	12.32	3.98	70	12.51	4	60	28.3%	-0.05 [-0.39, 0.30]
Linnanmäki 2020	25	18	27	23	16	33	26.4%	0.12 [-0.39, 0.63]
Raeissadat 2014	18.8	16	31	25.09	14	30	26.4%	-0.41 [-0.92, 0.10]
Thanasas 2011	0.68	0.13	14	1.15	0.21	14	18.9%	-2.61 [-3.66, -1.57]
**Subtotal (95% CI)**			**142**			**137**	**100.0%**	**-0.58 [-1.33, 0.16]**
Heterogeneity: Tau² = 0.48; Chi² = 23.30, df = 3 (P < 0.0001); I² = 87%								
Test for overall effect: Z = 1.54 (P = 0.12)								
**8.1.4 Function – (12 month follow-up)**								
Linnanmäki 2020	19	20	31	16	16	38	51.9%	0.17 [-0.31, 0.64]
Raeissadat 2014	21.82	18	31	26.84	18.05	30	48.1%	-0.27 [-0.78, 0.23]
**Subtotal (95% CI)**			**62**			**68**	**100.0%**	**-0.05 [-0.48, 0.39]**
Heterogeneity: Tau² = 0.03; Chi² = 1.55, df = 1 (P = 0.21); I² = 36%								
Test for overall effect: Z = 0.21 (P = 0.83)								
Test for subgroup differences: Chi² = 4.18, df = 3 (P = 0.24), I² = 28.3%								

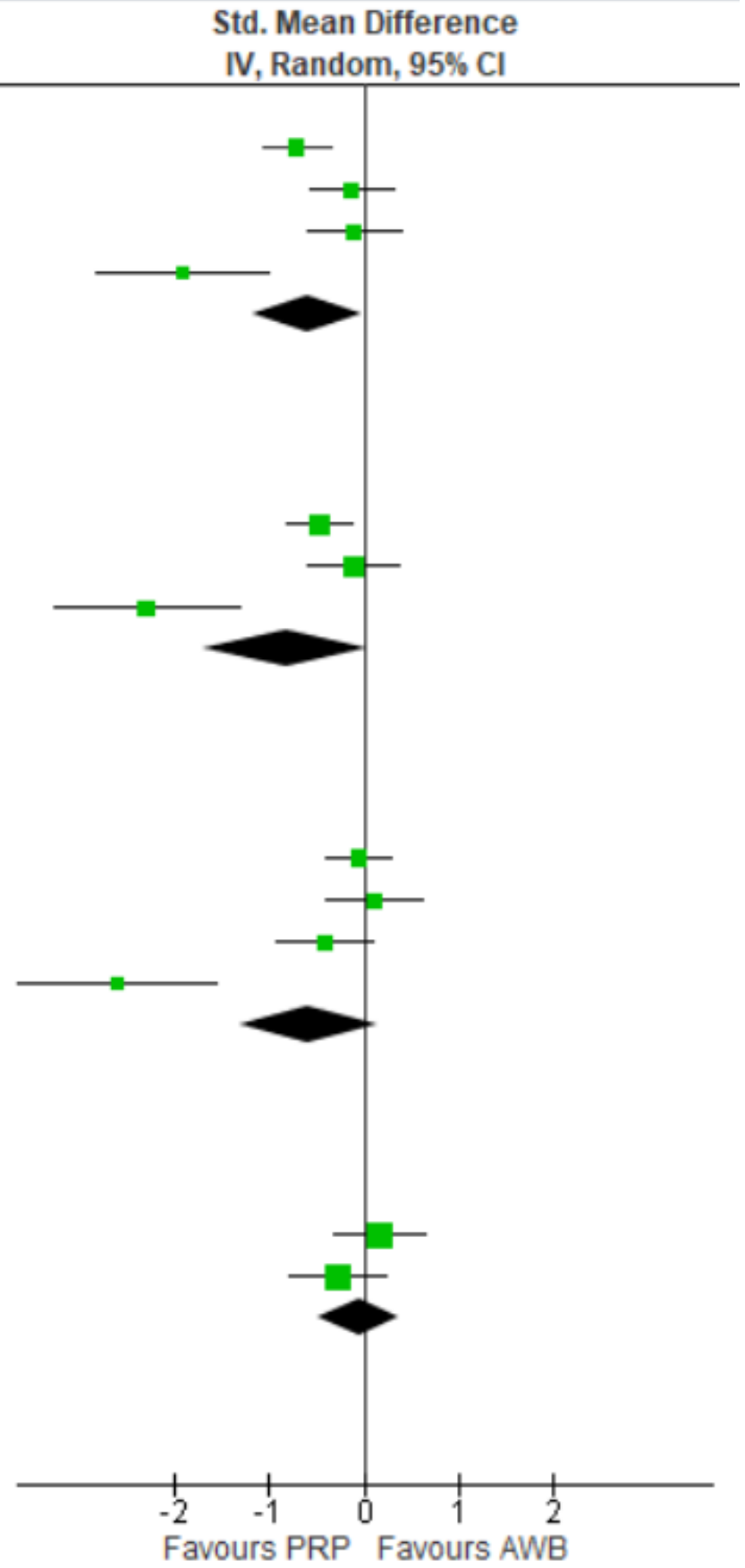

S1 - Functional outcome analysis comparing PRP and AWB at different follow-up periods (1, 3, 6, and 12 months); effects showed a trend favoring PRP in the early periods, with an effect approaching zero at 12 months. PRP: platelet-rich plasma; AWB: autologous whole blood.

Study or Subgroup	PRP Mean	PRP SD	PRP Total	AWB Mean	AWB SD	AWB Total	Weight	Mean Difference IV, Random, 95% CI
**9.1.1 Pain (VAS) – (1 month follow-up)**								
Linnanmäki 2020	5	2	40	4.7	1.9	40	32.2%	0.30 [-0.55, 1.15]
Raeissadat 2014	4.17	2.2	33	4.01	2.3	31	28.7%	0.16 [-0.94, 1.26]
Thanasas 2011	2.35	0.24	14	3.5	0.31	14	39.1%	-1.15 [-1.36, -0.94]
**Subtotal (95% CI)**			**87**			**85**	**100.0%**	**-0.31 [-1.43, 0.82]**
Heterogeneity: Tau² = 0.83; Chi² = 15.07, df = 2 (P = 0.0005); I² = 87%								
Test for overall effect: Z = 0.54 (P = 0.59)								
**9.1.2 Pain (VAS) – (2 month follow-up)**								
Linnanmäki 2020	4.2	2.2	30	4.1	2.1	35	49.0%	0.10 [-0.95, 1.15]
Raeissadat 2014	3.29	2.05	31	3.75	2.05	30	51.0%	-0.46 [-1.49, 0.57]
**Subtotal (95% CI)**			**61**			**65**	**100.0%**	**-0.19 [-0.92, 0.55]**
Heterogeneity: Tau² = 0.00; Chi² = 0.56, df = 1 (P = 0.46); I² = 0%								
Test for overall effect: Z = 0.50 (P = 0.62)								
**9.1.3 Pain (VAS) – (3 month follow-up)**								
Linnanmäki 2020	3.9	2.5	27	3.4	2.4	33	39.2%	0.50 [-0.75, 1.75]
Thanasas 2011	1.92	0.23	14	2.78	0.23	14	60.8%	-0.86 [-1.03, -0.69]
**Subtotal (95% CI)**			**41**			**47**	**100.0%**	**-0.33 [-1.63, 0.97]**
Heterogeneity: Tau² = 0.72; Chi² = 4.47, df = 1 (P = 0.03); I² = 78%								
Test for overall effect: Z = 0.49 (P = 0.62)								
**9.1.4 Pain (VAS) – (6 month follow-up)**								
Linnanmäki 2020	3.9	2.5	27	3.4	2.4	33	19.5%	0.50 [-0.75, 1.75]
Raeissadat 2014	2.91	2.47	31	3.41	2.13	30	21.6%	-0.50 [-1.66, 0.66]
Thanasas 2011	1.78	0.29	14	2.53	0.29	14	58.9%	-0.75 [-0.96, -0.54]
**Subtotal (95% CI)**			**72**			**77**	**100.0%**	**-0.45 [-1.12, 0.21]**
Heterogeneity: Tau² = 0.18; Chi² = 3.87, df = 2 (P = 0.14); I² = 48%								
Test for overall effect: Z = 1.33 (P = 0.18)								
Test for subgroup differences: Chi² = 0.28, df = 3 (P = 0.96), I² = 0%								

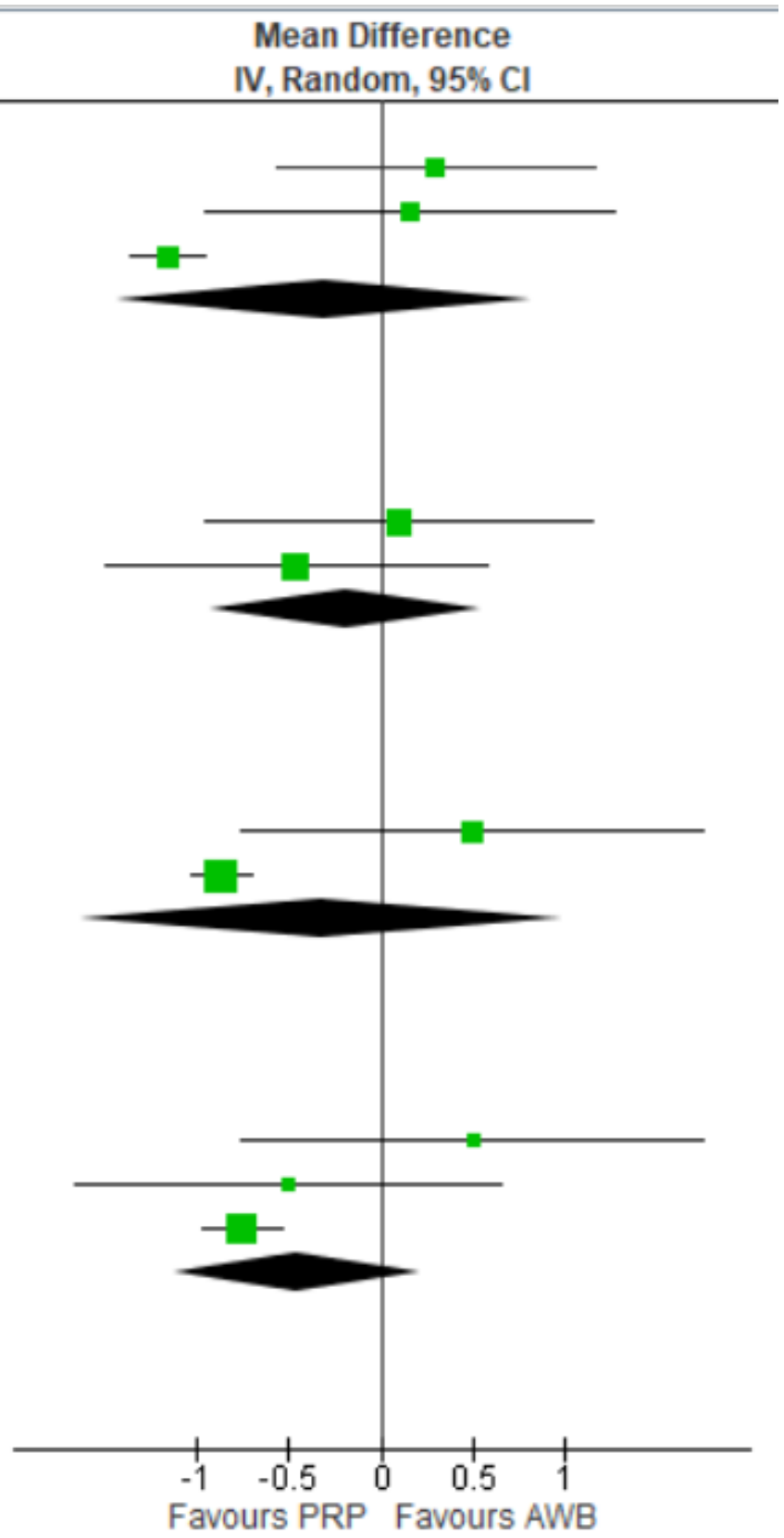

S2 – Pain outcome analysis comparing PRP and AWB at different follow-up periods (1, 2, 3, and 6 months): no significant differences were observed between the groups. Significant heterogeneity was found at 1 and 3 months, no heterogeneity at 2 months, and substantial heterogeneity at 6 months (48%). PRP: platelet-rich plasma; AWB: autologous whole blood.
